# Effects of Supplementing Zinc Magnesium Aspartate on Sleep Quality and Submaximal Weightlifting Performance, following Two Consecutive Nights of Partial Sleep Deprivation

**DOI:** 10.3390/nu16020251

**Published:** 2024-01-13

**Authors:** Chloe Gallagher, Victoria Austin, Kyle A. Dunlop, Jasmine Dally, Kyle Taylor, Samuel A. Pullinger, Ben J. Edwards

**Affiliations:** 1 Research Institute for Sport and Exercise Sciences, Liverpool John Moores University, Tom Reilly Building, Byrom Street Campus, Liverpool L3 3AF, UK; victoria97austin@gmail.com (V.A.); k.a.dunlop@ljmu.ac.uk (K.A.D.); jasdally7@gmail.com (J.D.); coach.kylewtaylor@gmail.com (K.T.); b.j.edwards@ljmu.ac.uk (B.J.E.); 2 Sport Science Department, Inspire Institute of Sport, Vidyanagar, Bellary 583275, India; samuel.pullinger@inspireinstituteofsport.com

**Keywords:** supplementation, dietary factors, micronutrients, muscle force output, individualised response, sleep restriction

## Abstract

**Purpose:** We examined whether supplementation of zinc magnesium aspartate (ZMA), while partially sleep deprived, was beneficial to sleep quality and subsequent morning (07:00 h) submaximal weightlifting. **Methods:** Using a double-blinded, randomized counterbalanced design, sixteen trained males were recruited and completed six sessions: (i) one repetition max (1 RM) for bench press and back squat; (ii) two familiarisation sessions; (iii) three conditions with 4 h sleep and either: ZMA, placebo (PLA), or NoPill control (NoPill). Submaximal exercise session consisted of three repetitions at 40, 60 and 80% of 1 RM for bench press and back squat. Average power (AP), average velocity (AV), peak velocity (PV), displacement (D) and time-to-peak velocity (tPV) were recorded using MuscleLab linear encoders. Data were analysed using a general linear model with repeated measures and linear correlation. **Results:** No significant main effect for condition was found for performance values or subjective ratings of fatigue. Main effect for “load” on the bar was found, where AP and tPV values increased with load (*p* < 0.05). No significant relationship between dose of zinc or magnesium ingested and change in performance for 80% 1 RM power-outputs was found. **Conclusion:** Supplementation of ZMA for two nights of partial sleep deprivation had no effect on sleep or subsequent morning performance.

## 1. Introduction

The use of supplements to meet nutritional recommendations in the diet is very common among the athletic population, with greater than 40% of athletes worldwide using some form of dietary supplement [1]. Global market forecasts for the dietary supplement market amounted to approximately EUR 101 billion in 2018, with growth predicted to reach EUR 278 billion by 2024 [2,3,4]. Mineral supplements are one of the most purchased products, likely due to the known importance of minerals for bodily functions [5,6]. Zinc (Zn), an essential trace mineral within the body, plays a key role in the central nervous system and as a signalling mediator between cells at inter- and intra-cellular levels [7]. When compromised, low Zn status can impact immune and cognitive functions as well as wound healing. Low levels of Zn may also impact hormone levels such as testosterone, which consequently affects muscle mass and strength [8]. Magnesium (Mg) is an important macro-mineral and co-factor in >300 chemical reactions and has a fundamental role as a physiological regulator in nervous and muscular systems [9,10]. In some populations of recreational athletes’, dietary consumption levels of both Zn and Mg are below the recommended daily allowance (RDA; [11]). This is exacerbated by increased excretion due to exercise, as greater urination and sweat loss can increase mineral requirements by 10–20% [12,13,14]. Zinc magnesium aspartate (ZMA) has therefore become a popular supplement for recreational and elite athletes which has led to several studies investigating the proposed benefits for health and sporting performance [8,11,13].

Partial sleep loss (a 2–3 h reduction of sleep per night compared to that habitually taken in a 24 h period) over several days is a common occurrence in athletic and non-athletic populations [15,16,17]. Within athletes, this is attributed to high training and competition demand whereby a session might involve early rising or retiring late at night. Combined with time zone transition disturbance, environmental and psychological factors, athletes are susceptible to achieving <7 h of sleep per night. This can have detrimental effects on motivation—an essential element for tasks requiring maximal or submaximal efforts [18,19]. The current literature investigating the effects of ZMA on sporting performance measures suggests that the combined effect of this supplement may help to promote anabolic factors by increasing levels of total and free testosterone and insulin-like growth factor 1 (IGF-1; [20]). Based on these proposed effects, supplementation may reduce skeletal muscle catabolism and result in greater power output and increased muscle mass [21]. Recently, linear encoder technology has been used in combination with multi-joint exercise protocols to better monitor and evaluate muscle force output [22]. The MuscleLab linear encoder provides a suitable tool to detect fluctuations for the three dimensions of muscle force output: average power, peak velocity and time-to-peak velocity (AP, PV and tPV, respectively) after interventions [23,24].

Recent bodies of evidence have suggested that micronutrients, such as Zn and Mg play a role in sleep and circadian regulation. A proposed mechanism in which they may promote sleep is through their roles in synthesis and function of sleep–wake neurotransmitters such as gamma-aminobutyric acid (GABA)—a receptor that supports sleep when activated [25]. Unlike populations whose Zn and Mg are below the recommended daily allowance to whom most of the literature investigates, athletes who are suffering from partial sleep deprivation (but otherwise are healthy with no sleep disorders and have a balanced diet) may experience improvements in sleep variables with ZMA supplementation [8]. Reductions in sleep latency and/or fragmentation within their restricted sleep window may result in reduced detriments to morning sporting (maximum and submaximal strength) and cognitive performance by supplementing ZMA pre-sleep. In the sport of weightlifting, heats are generally in the morning with finals timed in the evening. This can result in partial sleep restriction (PSR) before an event or training being particularly common. Acute ZMA supplementation (one or two nights) and subsequent sleep and performance in this population has received little attention, with most studies primarily recruiting clinical populations with pre-existing sleep disorders [26]. As the specific mechanisms of these micronutrients on sleep remain unclear, further work is required given the potential benefits of ZMA for general sleep health and performance. 

We aimed to investigate whether acute ZMA supplementation, when under conditions of PSR (4 h sleep per night, over two consecutive nights), improved (i) markers of objective and subjective sleep for two nights (via actigraphy and sleep questionnaires), (ii) morning muscle force output during multi-joint movements, and (iii) morning Stroop performance, compared to prescribing a placebo (PLA) or a control of no pill taken (NoPill). We hypothesized that ZMA would have no beneficial effects on (a) ‘sleep’ variables, (b) muscle force output, and (c) cognitive performance (Stroop task/test) in our chosen population of healthy male recreational athletes.

## 2. Materials and Method

### 2.1. Participants

Sixteen males as identified by sex and gender (mean ± SD: 22 ± 1.6 years; body mass: 76.4 ± 10.7 kg; body stature: 176.6 ± 5.2 cm; normative retiring and rising times: 23:34 ± 00:23 h:min and 08:02 ± 00:58 h:min, respectively), participated in the study. Sample size was determined using power calculation software (G*Power, version 3.1.9.6), based upon a large effect size of 0.8 for AP with a power of 0.80 and an α = 0.05 which determined a sample of 12 participants was required. Similar effect sizes of 0.90 for AP have been reported by Brotherton et al. (2019) [24] who employed similar techniques and study design. In line with our inclusion criteria, participants were recreationally active (as classified by the ‘Participant Classification Framework’; [27]), injury-free with no diagnosed sleep disorders, not completed shift work or travelled outside the local time zone in the past month and had ≥2 years of strength and weight-based training experience. Prior to participating in the study, participants were presented with an information sheet followed by a ‘Physical Activity Readiness Questionnaire’ (PARQ; [28]) and a written consent form. Verbal explanation of the experimental procedure was provided; this included the aims of the study, the possible risks associated with participation and the experimental procedures. Participants were assessed for circadian chronotype using the ‘Composite Morningness/Eveningness Questionnaire’ by Smith et al. (2015) [29]. The mean chronotype score on a 13–52-point scale was 32.6 ± 3.3, hence all participants were intermediate type. Questionnaires were also performed to assess sleep flexibility/rigidity (F/R) and languidity/vigour (L/V), with mean scores of 46.8 ± 3.7 (F/R) and 44.1 ± 5.4 (L/V), indicating more flexibility and languidity. Experimental procedures were approved by the Human Ethics Committee at Liverpool John Moores University and conducted in accordance with the ethical standards of the journal and complied with the Declaration of Helsinki.

### 2.2. Research Design

**Figure 1:**
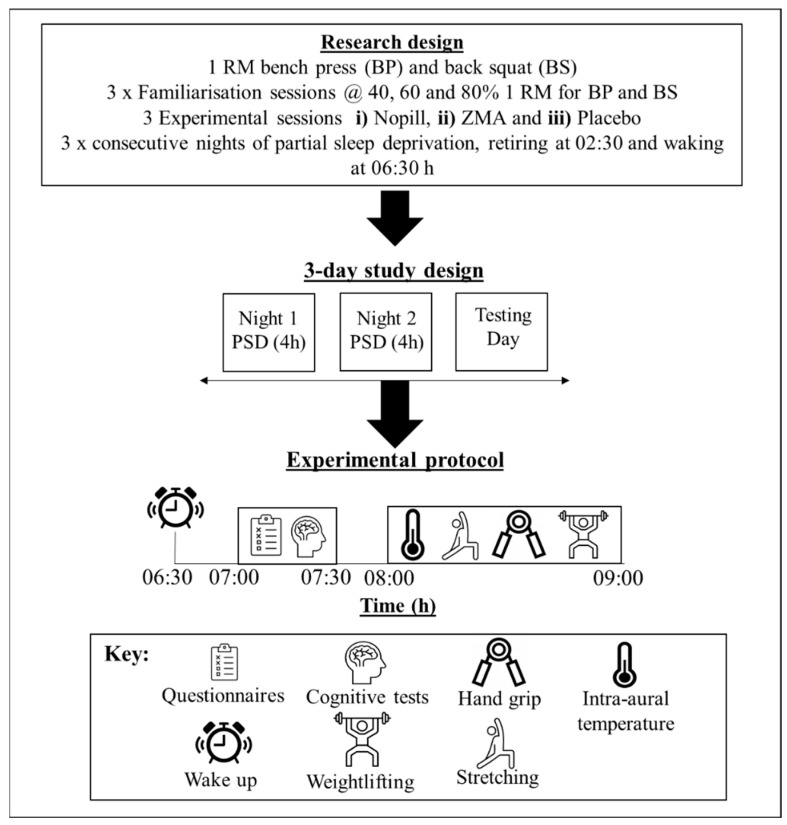
Schematic of experimental protocol. Participants followed the same procedures for each condition, with the addition of a ZMA supplement or placebo pill to be consumed at 01:00 h or no supplementation (NoPill). At 07:00 h, participants entered the laboratory and undertook the performance measures.

All participants were required to visit the laboratory on seven occasions (dry temperature of 19 °C, 35–45% humidity and a barometric pressure of 750–760 mmHg, respectively). Prior to attending the laboratory, participants completed a 5-day habitual food/fluid diary and weighed food intake, 7-day habitual sleep recording using actimetry (Motionwatch 8, CamnTech, Finstanton, UK), in addition to a sleep diary as a secondary measure (Table 1). The initial laboratory visits involved completion of 1 RM for bench press and back squat, followed by a further two visits for familiarisation sessions. Both familiarisations involved collection of participant height and mass, completion of questionnaires (Profile of Mood States (POMS); Stanford Sleepiness Scale; and sleep questions from the Liverpool Jet-lag questionnaire [30]); completion of exercise protocol which involved performing an active warm up and completing lifts at 40, 60 and 80% 1 RM for bench press and back squat (see Figure 1 and ‘measurements’ section for details). The remaining sessions consisted of three experimental conditions, involving two consecutive nights of sleep restriction (retiring at 02:30 and rising at 06:30 h) at the participant’s home before entering the laboratory at 07:00 h on the third day. Prior to bed, the participants either had three ZMA or PLA capsules or NoPill dependent on the condition. ZMA capsules contained zinc: 30 mg, magnesium: 450 mg, vitamin B6: 10.5 mg (Ph.D. Nutrition Ltd., Hull, UK) and placebo capsules were made in the department and contained maltodextrin (Sport supplements Ltd. t/a Bulk^TM^, Colchester, UK). Researchers and participants were blinded to the supplement schedule and pills were provided in a plastic bottle with instructions to consume with water. Both ZMA and placebo were lightly dusted with maltodextrin to create a similar taste, both had similar weight (0.8 g/capsule) and were 00 size. At the end of the experiment, the order of treatment was revealed to the researchers by an author (BE). Before experimental sessions, participants were asked to refrain from vigorous physical activity 24 h prior, during which time they also had to avoid any alcoholic or caffeine containing drinks. No food was to be consumed 1–2 h before experimental protocol, for the morning testing session and before sleep. In the hour before retiring to sleep participants were asked to refrain from watching television or use of their mobile devices and were also required to consume supplements provided if on the ZMA or PLA condition. To ensure recovery and to enable wash out for the ZMA between trials, there was at least a week between testing conditions for all participants. For testing to be conducted in a staggered manner, volunteers were closely matched based on 1 RM values. The experimental sessions were then counterbalanced in order of administration to minimise any potential learning effects [31], with a minimum of 72 h to ensure recovery between trials. All experiments were completed between the months of October and May (Autumn to Spring in the UK) with sunrise and sunset ranging from start to the end of the experiment being 05:37 to 07:29 h and 18:01 to 20:40 h, respectively. Testing was supposed to finish in February to ensure the individual’s exposure to sunlight in the mornings when entering the laboratories was <80 Lux. Unfortunately, due to COVID-19 restrictions, we had to extend the time frame.

### 2.3. Measurements

Prior to the main experimental laboratory sessions, 1 RM sessions determined each participant’s 1 RM percentages for incremental loads of 40, 60 and 80%, allowing a 5 min recovery between each effort. Familiarisation sessions reduced any learning effects and ensured the participants were physically capable and the risk of failed efforts during bench press and back squat were reduced. Following two consecutive nights of PSR, participants arrived at the laboratory at 07:00 h and after 30 min recordings of intra-aural temperature (Genius 1000, Mark 3, Sherwood, Nottingham, UK) were taken. Next, participants completed ratings of mood (Profile of Mood State questionnaire; [32]) and quality of sleep and sleepiness (Stanford Sleepiness Scale; [33]). Participants then undertook the Stroop test, where they were asked to read out their responses to words or colours for 45 s. This was filmed and the number of errors and total amount completed was recorded and analysed. A standardised active warm was performed on a cycle ergometer (Lode Corival, Furth, Germany) at 150 W for 5 min, followed by a series of dynamic movements which was repeated twice and involved squats (×10), lunges (5 each leg), single leg Romanian deadlifts (5 each leg) and press ups (×10). Post warm up, participants had three attempts at left and right grip strength, using a dynamometer (Takei KiKi Kogyo, Tokyo, Japan), and the highest value was recorded. To prepare for bench press and back squat, the force velocity linear encoder (Muscle Lab, Ergotest version 4010, Langensund, Norway) was attached to a 20 kg Olympic bar to measure displacement (D), average power (AP), peak velocity (PV), average velocity (AV), and time to peak velocity (tPV). Participants then completed bench press and back squat at 40, 60 and 80% of their 1 RM, completing three repetitions for each incremental load, with a 2 min rest between each incremental load. Submaximal lifts were recorded using force transducers; each session performed in the same order of muscle magnitude. Between each set, while resting, participants gave a value for rate of perceived exertion (RPE, 0–10 cm visual analogue scale), breathing and muscle fatigue on the 6–20 scale Borg scale [34]. Values were also given for rating of effort for submaximal lifts completed, presented as a visual analogue scale (VAS: 0–10, where zero is no effort and 10 is maximal [35]). To analyse all variables collected using the force transducer, we took the highest of the three AP outputs. Associated AV, PV, D and tPV values were used for subsequent analysis for each mass on the bar for both bench press and back squat, respectively (see Figure 1). In between experimental conditions, participants were under ‘normal living’ conditions.

### 2.4. Statistical Analysis

The Statistical Package for the Social Sciences (SPSS IBM) version 28, for Windows was used. Differences between conditions were evaluated using a general linear model with repeated measures, within subject factor for condition (three levels), within subject factor for ‘load on bar’ (three levels) or night (two levels) and interaction between all three variables. To correct violations of sphericity, the degrees of freedom were corrected in a normal way, using Huynh–Feldt (ε > 0.75) or Greenhouse–Geisser (ε < 0.75) values for ε, as appropriate. Graphical comparisons between means and Bonferroni pairwise comparisons were made where main effects were present. The α level of statistical significance was set at *p* < 0.05. Effect sizes (ES) were calculated from the ratio of the mean difference to the pooled standard deviation. The magnitude of the ES was classified as trivial (≤0.2), small (>0.2–0.6), moderate (>0.6–1.2), large (>1.2–2.0) and very large (>2.0) based on guidelines from Batterham and Hopkins (2006) [36]. Pearson correlations were conducted to explore individualised responses to Zn and Mg and performance. The results are presented as the mean ± standard deviation (SD) throughout the text unless otherwise stated. Ninety-five percent confidence intervals (CI) are presented where appropriate as well as the mean difference between pairwise comparisons.

## 3. Results

### 3.1. Performance Measures (Measured at 07:30 h)

**Figure 2:**
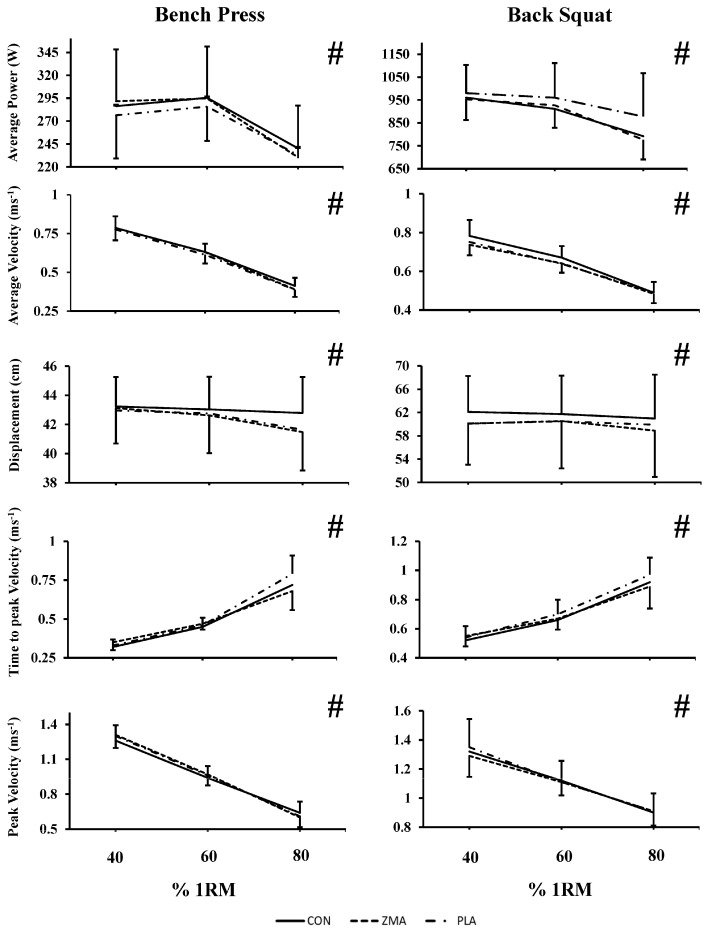
Mean and 95% CI values of each performance variable for morning (07:30 h) bench press and back squat at 40, 60 and 80% 1 RM loads for the three experimental conditions. # denotes main effect for load as shown by Bonferroni pairwise comparisons (*p* < 0.05).

Mean ± SD values and the results from the ANOVA statistical analysis are displayed in Table 2 and Table 3. Statistical significance of the results can be seen in Figure 2.

### 3.2. Grip Strength (Left and Right Hand)

There was no significant effect of condition on maximal left- or right-hand grip strength values (*p* = 0.887, *p* = 0.928, respectively; see Table 2). 

### 3.3. Bench Press

There was no significant main effect of condition for all bench press performance variables (AP, AV, D, PV and tPV; see Table 2). However, there was a significant main effect of ‘load’ for all bench press variables measured (see Table 2). For AV, D and PV, values were highest at 40% of 1 RM (0.78 ± 0.03 ms^−1^, 43.10 ± 1.14 cm, 1.29 ± 0.06 ms^−1^, respectively) and lowest at 80% 1 RM (0.40 ± 0.02 ms^−1^, 41.96 ± 1.24 cm, 0.62 ± 0.04 ms^−1^, respectively). Whereas tPV was significantly lower at 40% 1 RM (0.33 ± 0.02 ms^−1^) and highest at 80% 1 RM (0.71 ± 0.06 ms^−1^). As anticipated, there was a corresponding significant main effect of ‘load’ on subjective effort and RPE values (*p* < 0.05). At 40% of 1 RM load there was lower subjective values (Effort: 3.1 ± 0.3; RPE: 9.0 ± 0.3; RPE Breathing: 7.6 ± 0.3; RPE Muscle Fatigue: 8.5 ± 0.3) whereas 80% of 1 RM elicited the highest (Effort: 7.6 ± 1.2; RPE: 15.1 ± 0.5; RPE Breathing: 11.6 ± 0.6; RPE Muscle Fatigue: 15.1 ± 0.5). There was no significant interaction of ‘condition and load’ for any variables, such that values across all conditions at both time points for the three loads rose or fell in the same manner (see Figure 2).

### 3.4. Back Squat

There was no significant main effect for condition for all back squat performance variables (see Figure 2 and Table 2). However, there was a significant main effect of ’load‘ on all back squat variables, apart from displacement (*p* = 0.173; see Table 2). As expected, AP, AV and PV were highest at 40% of 1 RM load (964.55 ± 46.29 W; 0.76 ± 0.03 ms^−1^; 1.32 ± 0.07 ms^−1^, respectively) and lowest at 80% of 1 RM load (815.36 ± 53.48 W; 0.49 ± 0.03 ms^−1^; 0.90 ± 0.05 ms^−1^, respectively). Whereas tPV was significantly lower at 40% 1 RM (0.54 ± 0.03 ms^−1^) and highest at 80% 1 RM (0.93 ± 0.06 ms^−1^). As anticipated, there was a corresponding significant main effect of ‘load’ on subjective effort and RPE values (*p* < 0.05). At 40% of 1 RM load there was lower subjective values (Effort: 3.5 ± 0.2; RPE: 9.1 ± 0.3; RPE Breathing: 8.2 ± 0.4; RPE Muscle Fatigue: 8.7 ± 0.3), whereas 80% of 1 RM elicited the highest (Effort: 7.9 ± 0.3; RPE: 15.9 ± 0.6; RPE Breathing: 12.9 ± 0.6; RPE Muscle Fatigue: 15.6 ± 0.6). There was no significant interaction of ‘condition and load’ for any variables, such that values across all conditions at both time points for the three loads rose or fell in the same manner (see Figure 1).

## 4. Physiological and Psychological Variables (Measured at 07:30 h) 

### 4.1. Intra-Aural Temperature, Tiredness and Alertness

There was no significant main effect of condition on intra-aural temperature or subjective ratings of tiredness and alertness (*p* > 0.05, see Table 3), indicating that supplementing ZMA did not have a significant effect on core temperature values or subjective ratings of alertness and tiredness compared to PLA and NoPill conditions. 

### 4.2. Profile of Mood State

There was no significant effect of condition for all mood profiles (anger, calm, confused, depressed, fatigue, happiness), see Table 3.

### 4.3. Stroop (Word–Colour Interference Test)

There was a significant main effect of condition (*p* = 0.023; see Table 3) for the word–not colour interference for total score. Pairwise comparisons show that the NoPill condition achieved the lowest total score when compared to the ZMA condition (98.1 ± 3.7; 95% CI: 90.2–106.1, ES = 0.41). Whereas the highest total score was achieved in the PLA condition (107.5 ± 3.7; 95% CI: 99.5–115.4, ES = 0.26). However, there was no significant effect of condition for word–not colour errors, colours–not words errors and colours–not words totals (*p* = 0.836; *p* = 0.371; *p* = 0.454, respectively; see Table 3).

## 5. Measures of Sleep

***Actigraphy variables:*** There was no significant main effect of condition for any actigraphy variables (actual sleep time, sleep efficiency, sleep latency and fragmentation index; see Table 4). There was no significant main effect between nights of partial sleep deprivation for any actigraphy variables (*p* > 0.05). No significant interactions of ‘condition and night’ were identified (see Table 4).

***Stanford Sleepiness:*** There was no significant main effect of condition on subjective sleepiness ratings (*p* = 0.503).

Correlations between dose of mineral and habitual intake/kg body mass and percent change in performance (average power at 80% 1 RM for bench press and back squat.

**Figure 3:**
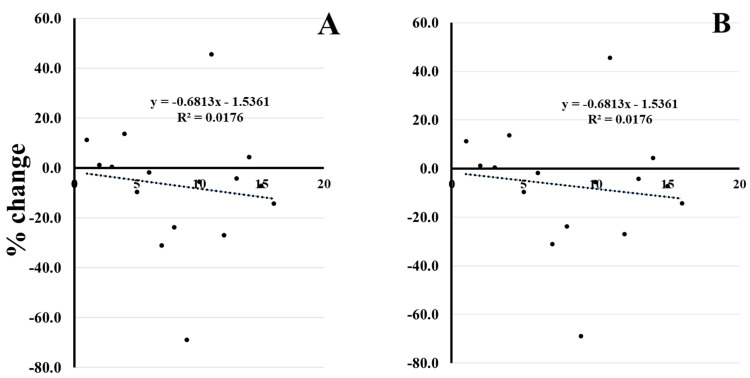

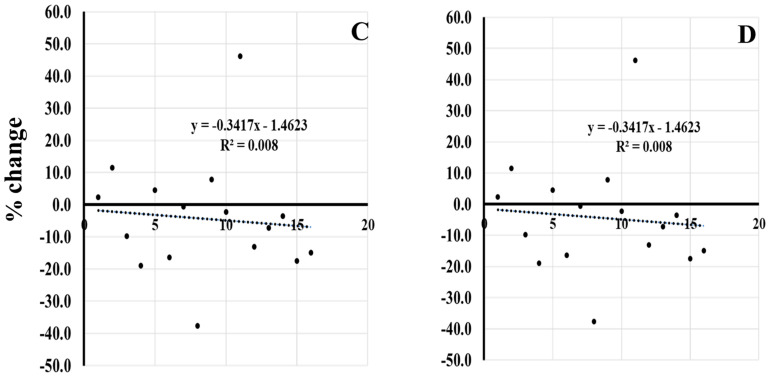
Correlation of change in average Power 80%1×RM expressed Zn Intake/kg body mass for (**A**) ZMA vs. no pill or for ZMA vs. no pill (**B**). Or Mg Intake/kg body mass for (**C**) ZMA vs. no pill or for ZMA vs. no pill (**D**).

Pearson correlation showed no significant linear relationship between individual dose of zinc or magnesium habitually ingested each day (both in the participants’ diet and through the consumption of the tablets) and change in performance for 80% 1 RM average power outputs for bench press and back squat between conditions (PLA, ZMA and NoPill respectively; Figure 3).

## 6. Discussion

The main finding of the study is that ZMA supplementation for two nights of consecutive PSR in recreationally active males who already meet the RDA for the micronutrients in question, does not improve: (a) sleep (as measured by via actigraphy and sleep questionnaires), (b) muscle force output during multi-joint movements, or (c) subsequent morning cognitive function (colour–word interference test; [37]). Research into the use of ZMA and gross muscular maximal or submaximal performance is scarce and even more so regarding the effects on sleep [8,11,13]. We adopted a pragmatic approach involving a protocol that assessed the effects of an acute sleep disruption (commonly found in athletes), often associated with travel to competition or training [15,38]. This procedure has been utilised by others [16,24,39]. 

Comparison between our findings and Wilborn and colleagues (2004) [13] are consistent, whereby we had a similar protocol (ZMA vs. placebo supplementation, submaximal weightlifting), provided similar quantities of Mg, Zn and B6 (450 mg of Mg, 30 mg of Zn and ~11 mg of B6) and recruited recreationally strength-trained males. Opposed to our randomised, counterbalanced approach with double-blinded design, Wilborn et al. (2004) [13] used a repeated measures design with others using independent groups with smaller sample sizes [8,11]. Previous studies that have investigated ZMA supplementation state that dietary analysis was conducted but only Moëzzi et al. (2013) [8] provided data for percent of macronutrient contributions, with Brilla and Conte (2000) [11] stating that participants met the RDA for Zn, Mg and B6. In the current study, habitual dietary intake of participants was recorded for 5 days, whereby proportions of macronutrients and micronutrients were accounted for (see Table 1). Duration of supplementation also differed between studies, with ZMA either administered chronically (i.e., 7 to 8 weeks and with measures pre and post intervention) or acutely, like the current study. It is arguable that our supplementing period was too short for effects to be observable. Some studies that employed supplement periods that extend >8 weeks have shown markers of improved sleep duration and efficiency [40]. Lastly, we utilised a study design that incorporated a NoPill group in conjunction with the ZMA and PLA group, unlike other studies. This was to ensure that any placebo effect is accounted for and that the true potential effect of the supplement can be established. 

Contrary to the findings of the current study, Zn and Mg have previously been shown to improve sleep parameters in healthy elderly populations and those who have poor dietary intake, such that they have micronutrient deficiencies [7,40]. To the best of our knowledge, the use of ZMA to aid sleep following PSR has never been investigated in individuals without pre-existing sleep disorders and/or nutrient deficiencies. In the current study measures of sleep, via objective and subjective assessments, we found no differences between conditions for all sleep variables. It was hypothesised that ZMA improves sleep by a decrease in sleep latency and an increase in the quantity of slow wave sleep [41,42]. Magnesium may act to increase the activation of GABA neurotransmission which contributes to improvement in sleep architecture, particularly slow-wave sleep which is associated with restorative sleep [20,21]. However, we found no evidence in improvements in sleep variables in our measures. As we did not use polysomnography, we are unable to report information on sleep stages, but future research should ensure the use of polysomnography to confirm or deny this hypothesis. 

It has previously been shown that submaximal lifts measured using linear encoders are sensitive to partial sleep deprivation [24]. Despite this, we reported no significant effect of condition, with no differences between ZMA, PLA or NoPill for maximal grip strength values or submaximal muscle strength measures for bench press or back squat (AP, AV, D, PV and tPV; see Table 2); these are movements that are more transferable to athletic performance [23,43]. This agrees with Moëzzi et al. (2013) [8] and Wilborn et al. (2004) [13] who found no improvements in the ZMA condition compared to PLA after 7- and 8-weeks training with evening supplementation, respectively. In contrast, Brilla and Conte (2000) reported ~10% greater torque and ~12–15% increase in power output for hamstring and quadriceps after 8 weeks supplementation, when ZMA was compared to PLA. These improvements were attributed to an increase in testosterone and IGF-1; thus, indicating a potentially ergogenic effect. It is important to highlight that the participants in the ZMA group had body mass values ~3 kg heavier than the control group [11]. Although no statistics reported the change in body mass pre to post, this may potentially explain the change in strength for the PLA group. Lean body mass would need to be assessed, given that lean body muscle is associated with greater power and strength qualities [44]. Alternatively, other studies [13] have previously matched participants based on fat-free mass to allow for a more homogeneous sample. Whilst all three studies employed differing allocation techniques, the current study’s crossover design allows each participant to be their own control and minimises potential bias. It should also be noted that the differences in strength protocols used (isolated movements vs. compound movements) may explain the discrepancies between the studies.

A significant main effect for ‘load’ was present for all bench press and back squat output variables (AP, D, AV, PV, tPV; see Table 2). From 40 to 80% 1 RM, where there was greater load against the movement, tPV increased yet PV, AV, AP (See Figure 2) were highest when the load on bar was lowest (40% 1 RM). Additionally, ratings of subjective effort and RPE values increased in line with the increasing load on the bar. These findings are consistent with the fundamental force–velocity properties of skeletal muscle and have been demonstrated previously during submaximal loads using MuscleLab linear encoders and force platforms during complex movements [23,45]. In agreement with previous research, tPV increased under greater resistance as it takes longer for the participant to generate and produce power to execute the movement. Interactions of ‘load and condition’ were not present for any performance variables. 

To assess for individual differences in relation to a dose response for supplementing ZMA, we conducted correlations between mineral dose and habitual intake and accounted for body mass (kg) and change in performance (AP at 80% 1 RM for bench press and back squat). Linear corelation showed no relationship between dose of Zn or Mg ingested (both in the participants diet and throughout the consumption of the supplements) and change in performance for 80% 1 RM power outputs (*p* > 0.05; Table 1). Even though current literature on this topic is scarce, it appears likely that ZMA does not produce strength-related benefits. It has been previously reported that when zinc intake is greater, there is an increase in the transport rate across the blood–brain barrier, but this begins to plateau at larger quantities [46]. Therefore, the nutritional status of each participant would in theory determine the supplemental uptake and thus the effect of the supplement on the individual.

Given the large cognitive component that is associated with ‘high-skilled’ movements (e.g., bench press and to a greater extent due to its more complex movement back squat), it is reasonable to suggest that mood state is a major factor that influences motivation and thus weightlifting performance [47,48]. This theory is supported by research that found that total resistance workload was reduced in partially sleep-deprived states (<6 h), but that this effect could be reversed with the ingestion of caffeine to restore alertness [49]. Zn and Mg supplementation, within non-athletic populations has also been previously shown to improve mood states, yet unlike caffeine, the mechanisms and understanding are still to be fully understood [50,51,52,53]. Further, no differences were reported for intra-aural temperature and subjective ratings of alertness and tiredness (see Table 3). Whilst these psychological components appear to underline detriments in muscular performance, our findings report no differences in mood states between conditions. To assess cognitive performance, we employed the Stroop test which is considered the gold standard for evaluating attentional measures. Our findings report a significant main effect of condition for the word–colour interference test (*p* = 0.023; see Table 3). Where the NoPill condition achieved the lowest total score (number of answers) for response of words with no change in number of errors when compared to ZMA (see Table 3). The highest total score was achieved in the PLA condition (see Table 3), further demonstrating that ZMA did not influence cognitive ability. This finding supports the use of a NoPill condition ensuring that any placebo effect is accounted for and that the true potential effect of ZMA can be established.

### Limitations

Actimetry may lack the sensitivity to detect change, particularly sleep latency, due to the device being unable to distinguish the difference between movement of the wrist during sleep and general non-movement [54]. Polysomnography would perhaps offer a greater level of accuracy needed to detect meaningful change. The significant effect of displacement for load indicates that ‘form’ was lost as the load on the bar increased, despite participant familiarisation session. Therefore, thought should be given as to how form can be retained such that it does not affect other Muscle Lab variables and the lift can be ‘standardised’. 

## 7. Conclusions

Our most important outcome was that ZMA did not improve markers of sleep quality, cognitive function, or gross muscular performance during a subsequent morning submaximal weightlifting session after two nights of PSR when compared to a PLA or a NoPill supplementation condition. 

### Practical Implications and Future Research

To our knowledge this is the only study to investigate partial sleep restriction on submaximal weightlifting performance relating to ZMA ingestion. The current findings may provide important recommendations and interventions for athletes who have high training/competition demands and are facing partial sleep restriction. Based on our results, the effectiveness of acute (two nights) ZMA supplementation in a healthy population who are not deficit of Zn, Mg or B6 is weak. Further work should investigate the mechanisms of ZMA during sleep using polysomnography, which would provide greater insight into optimal nap duration and the effectiveness of a nap on performance. Future research should also investigate chronic supplementation (4–8 weeks) as this may allow for a greater concentration of serum Zn/Mg status which could alter findings. Venous blood sampling would be integral to establishing both, post-supplement mineral serum and ‘pre’ habitual Zn/Mg status. Differentiating habitually high Zn/Mg consumers from low consumers may offer an insight as to whether ZMA affects these groups to different extents. 

## Figures and Tables

**Table 1:** Habitual food/fluid diary and weighed food intake.

Habitual Average Intake	Mean ± SD
Energy (kcal)	2256 ± 534
Carbohydrate (g)	238 ± 61
Carbohydrate (g/kg)	3 ± 1
Fat (g)	71 ± 20
Fat (g/kg)	1 ± 0
Protein (g)	146 ± 56
Protein (g/kg)	2 ± 1
Zinc (mg)	12
Magnesium (mg)	410
Vitamin B6 (mg)	3
**Habitual Sleep Variables**	
Normative retiring (h:mm)	23:50
Normative waking (h:mm)	08:02

Mean ± SD values for 5-day habitual food/fluid diary and weighed food intake, 7-day habitual sleep recording using actimetry (Motionwatch 8, CamnTech).

**Table 2:** F values, *p* values and effect size (ES) for all performance variables measured in this study (‘average power’, ‘average velocity’, ‘displacement’, ‘peak velocity’, ‘time to peak velocity’, and rating of perceived exertion (RPE) and visual analogue scale (VAS) for both bench press and back squat.

Variable	Significance Condition	Significance of Load	Interactions (COND and LOAD)
**Grip Strength (N)**			
Left	F_1.6, 23.4_ = 0.07 (*p* = 0.887), ES = 0.007		
Right	F_2.0, 30.0_ = 0.08 (*p* = 0.928), ES = 0.009		
**Bench Press**			
Average power (W)	F_1.9, 28.6_ = 0.43 (*p* = 0.645), ES = 0.042	**F_1.3, 20.1_ = 12.99 (*p* < 0.0005), ES = 0.397**	F_2.2, 32.5_ = 0.85 (*p* = 0.447)
Displacement (cm)	F_2.0, 30.0_ = 0.77 (*p* = 0.472), ES = 0.062	**F_2.0, 30.0_ = 7.46 (*p* = 0.002), ES = 0.418**	F_3.3, 49.1_ = 1.14 (*p* = 0.345)
Average velocity (ms^−1^)	F_2.0, 30.0_ = 0.45 (*p* = 0.644), ES = 0.007	**F_1.1, 16.4_ = 174.99 (*p* < 0.0005), ES = 0.928**	F_2.4, 35.5_ = 0.47 (*p* = 0.473)
Peak velocity (ms^−1^)	F_1.6, 24.1_ = 0.13 (*p* = 0.839), ES = 0.001	**F_1.2, 17.2_ = 210.92 (*p* < 0.0005), ES = 0.940**	F_2.3, 34.4_ = 2.35 (*p* = 0.104)
Time to peak velocity (s)	F_2.0, 30.0_ = 0.79 (*p* = 0.464), ES = 0.078	**F_1.1, 16.1_ = 31.60 (*p* < 0.0005), ES = 0.742**	F_1.8, 27.1_ = 2.81 (*p* = 0.083)
Perceived effort (VAS, 0–10 cm)	F_1.9, 26.7_ = 0.07 (*p* = 0.925)	**F_1.2, 17.9_ = 244.28 (*p* < 0.0005)**	F_2.4, 33.8_ = 0.46 (*p* = 0.670)
RPE (6–20)	F_2.0, 30.0_ = 0.68 (*p* = 0.514)	**F_1.3, 20.0_ = 147.85 (*p* < 0.0005)**	F_2.2, 32.2_ = 0.70 (*p* = 0.517)
RPE Breathing (6–20)	F_1.8, 27.5_ = 3.13 (*p* = 0.063)	**F_1.3, 20.0_ = 63.07 (*p* < 0.0005)**	F_3.1, 46.5_ = 1.00 (*p* = 0.401)
RPE Muscle fatigue (6–20)	F_1.4, 20.8_ = 1.68 (*p* = 0.203)	**F_1.5, 22.3_ = 179.52 (*p* < 0.0005)**	F_2.6, 38.3_ = 0.432 (*p* = 0.701)
**Back Squat**			
Average power (W)	F_1.3, 19.7_ = 0.83 (*p* = 0.406), ES = 0.062	**F_1.4, 21.0_ = 32.29 (*p* < 0.0005), ES = 0.707**	F_2.2, 32.4_ = 1.18 (*p* = 0.324)
Displacement (cm)	F_1.4, 21.4_ = 1.51 (*p* = 0.241), ES = 0.094	F_1.4, 21.1_ = 1.97 (*p* = 0.173), ES = 0.091	F_2.3, 34.9_ = 0.45 (*p* = 0.671)
Average velocity (ms^−1^)	F_2.0, 30.0_ = 0.89 (*p* = 0.421), ES = 0.050	**F_1.1, 16.4_ = 86.64 (*p* < 0.0005), ES = 0.858**	F_2.0, 29.9_ = 0.74 (*p* = 0.485)
Peak velocity (ms^−1^)	F_1.9, 28.7_ = 0.13 (*p* = 0.872), ES = 0.003	**F_1.2, 18.6_ = 40.17 (*p* < 0.0005), ES = 0.766**	F_2.2, 33.3_ = 0.18 (*p* = 0.858)
Time to peak velocity (s)	F_2.0, 30.0_ = 0.50 (*p* = 0.609), ES = 0.059	**F_1.2, 17.5_ = 73.61 (*p* < 0.0005), ES = 0.839**	F_2.2, 32.4_ = 1.00 (*p* = 0.385)
Perceived effort (VAS, 0–10 cm)	F_2.0, 28.0_ = 0.11 (*p* = 0.893)	**F_1.3, 18.0_ = 191.7 (*p* < 0.0005)**	F_2.1, 30.1_ = 0.45 (*p* = 0.657)
RPE (6–20)	F_2.0, 29.2_ = 0.89 (*p* = 0.418)	**F_1.2, 18.4_ = 192.70 (*p* < 0.0005)**	F_2.6, 38.7_ = 2.20 (*p* = 0.112)
RPE Breathing (6–20)	F_1.8, 27.6_ = 3.33 (*p* = 0.054)	F_1.2, 18.4_ = 72.65 (*p* = 0.060)	F_3.4, 51.4_ = 1.65 (*p* = 0.184)
RPE Muscle fatigue (6–20)	F_1.3, 19.1_ = 2.71 (*p* = 0.109)	F_1.3, 18.9_ = 206.09 (*p* < 0.0005)	F_4.0, 60.0_ = 1.02 (*p* = 0.405)

**Bold** indicates significant (*p* < 0.05); underline indicates a trend (0.1 < *p* > 0.05).

**Table 3:** Mean ± SD, F values and *p* values for all physiological and psychological variables measured in the study (temperature, tiredness, alertness, Profile of Mood States (POMS), word and colour interference test).

Variables	NOPILL	ZMA	PLA	Significance Condition
Intra-aural temperature (°C)	35.7 ± 1.1	35.6 ± 1.2	35.6 ± 1.1	F_2.0, 30.0_ = 0.09 (*p* = 0.918)
Tiredness (0–10 VAS)	8.0 ± 3.2	7.5 ± 3.4	7.2 ± 4.2	F_2.0, 30.0_ = 0.51 (*p* = 0.604)
Alertness (0–10 VAS)	3.2 ± 2.2	3.8 ± 2.3	3.8 ± 2.9	F_1.6, 23.9_ = 0.56 (*p* = 0.542)
Stanford Sleepiness	4.4 ± 1.4	4.1 ± 1.3	4.2 ± 1.4	F_1.5, 21.8_ = 0.61 (*p* = 0.503)
Mood State–Vigour	3.0 ± 2.7	3.6 ± 3.3	3.4 ± 3.1	F_1.5, 22.5_ = 0.39 (*p* = 0.624)
Mood State–Anger	1.8 ± 1.8	1.0 ± 2.3	1.9 ± 2.7	F_1.9, 28.5_ = 1.31 (*p* = 0.284)
Mood State–Tension	0.8 ± 1.3	0.6 ± 0.9	0.6 ± 0.7	F_1.5, 22.2_ = 0.30 (*p* = 0.681)
Mood State–Calm	5.8 ± 3.2	5.6 ± 4.1	6.6 ± 4.1	F_1.6, 24.3_ = 0.95 (*p* = 0.390)
Mood State–Happiness	4.1 ± 3.1	4.0 ± 4.2	4.3 ± 3.4	F_1.8, 26.6_ = 0.04 (*p* = 0.948)
Mood State–Confusion	1.9 ± 2.8	1.6 ± 1.5	1.9 ± 2.1	F_2.0, 30.0_ = 0.19 (*p* = 0.831)
Mood State–Depression	1.7 ± 1.9	1.5 ± 1.7	1.0 ± 1.4	F_2.0, 30.0_ = 1.25 (*p* = 0.302)
Mood State–Fatigue	8.8 ± 3.8	8.7 ± 3.8	8.3 ± 5.3	F_1.3, 19.9_ = 0.08 (*p* = 0.842)
STROOP (Colours/NotW/TOTAL)	58.1 ± 9.3	56.6 ± 11.2	60.6 ± 16.4	F_1.9, 28.2_ = 0.80 (*p* = 0.454)
STROOP (Colours/NotW/ERROR)	1.8 ± 1.6	2.4 ± 2.4	1.6 ± 1.2	F_1.4, 21.6_ = 0.96 (*p* = 0.371)
STROOP (Words/NotC/TOTAL)	98.1 ± 14.9	104.3 ± 12.5	107.5 ± 14.9	**F_2.0, 30.0_ = 4.28 (*p* = 0.023)**
STROOP (Words/NotC/ERROR)	0.9 ± 1.0	0.7 ± 0.9	0.8 ± 1.3	F_1.8, 27.0_ = 0.13 (*p* = 0.863)

**Bold** values indicate significant figures (*p* < 0.05).

**Table 4:** Mean ± SD, F values and *p* values for all actimetry variables measured in the study.

Actimetry Variables	NoPill		ZMA		PLA		Significance Condition	Significance Night	Significance Interaction
	N1	N2	N1	N2	N1	N2			
Actual sleep time (h:mm)	3:55 ± 0:22	3:51 ± 0:21	3:52 ± 0:15	3:42 ± 0:58	3:53 ± 0:15	4:00 ± 0:17	F_2.0, 30.0_ = 2.69 (*p* = 0.08)	F_1.0, 15.0_ = 0.11 (*p* = 0.742)	F_1.4, 21.1_ = 0.73 (*p* = 0.449)
Sleep latency (h:mm)	0:14 ± 0:14	0:12 ± 0:11	0:12 ± 0:15	0:13 ± 0:11	0:08 ± 0:10	0:10 ± 0:10	F_2.0, 30.0_ = 2.44 (*p* = 0.105)	F_1.0, 15.0_ = 0.00 (*p* = 0.985)	F_1.6, 24.0_ = 0.440 (*p* = 0.606)
Sleep efficiency (%)	80.7 ± 9.2	78.3 ± 16.8	79.7 ± 12.4	79.2 ± 8.3	76.2 ± 12.2	77.3 ± 19.1	F_1.3, 19.7_ = 0.45 (*p* = 0.559)	F_1.0, 15.0_ = 0.06 (*p* = 0.809)	F_1.3, 19.4_ = 0.31 (*p* = 0.644)
Fragmentation Index (%)	23.5 ± 15.1	22.8 ± 12.0	20.6 ± 12.4	24.3 ± 14.2	31.3 ± 15.1	28.7 ± 14.0	F_1.5, 22.6_ = 2.23 (*p* = 0.141)	F_1.0, 15.0_ = 0.00 (*p* = 0.969)	F_2.0, 30.0_ = 1.27 (*p* = 0.296)

Underline indicates a trend (0.05 < *p* < 0.1).

## Data Availability

Data is contained within the article.
